# MulinforCPI: enhancing precision of compound–protein interaction prediction through novel perspectives on multi-level information integration

**DOI:** 10.1093/bib/bbad484

**Published:** 2024-01-04

**Authors:** Ngoc-Quang Nguyen, Sejeong Park, Mogan Gim, Jaewoo Kang

**Affiliations:** Department of Computer Science and Engineering, Korea University, 02841, Seoul, Korea; Department of Computer Science and Engineering, Korea University, 02841, Seoul, Korea; AIGEN Sciences, 04778, Seoul, Korea; Department of Computer Science and Engineering, Korea University, 02841, Seoul, Korea; Department of Computer Science and Engineering, Korea University, 02841, Seoul, Korea; Interdisciplinary Graduate Program in Bioinformatics, Korea University, 02841, Seoul, Korea; AIGEN Sciences, 04778, Seoul, Korea

**Keywords:** compound–protein interaction, transfer learning, cross-attention, 3D geometric information, multi-level information

## Abstract

Forecasting the interaction between compounds and proteins is crucial for discovering new drugs. However, previous sequence-based studies have not utilized three-dimensional (3D) information on compounds and proteins, such as atom coordinates and distance matrices, to predict binding affinity. Furthermore, numerous widely adopted computational techniques have relied on sequences of amino acid characters for protein representations. This approach may constrain the model’s ability to capture meaningful biochemical features, impeding a more comprehensive understanding of the underlying proteins. Here, we propose a two-step deep learning strategy named MulinforCPI that incorporates transfer learning techniques with multi-level resolution features to overcome these limitations. Our approach leverages 3D information from both proteins and compounds and acquires a profound understanding of the atomic-level features of proteins. Besides, our research highlights the divide between first-principle and data-driven methods, offering new research prospects for compound–protein interaction tasks. We applied the proposed method to six datasets: Davis, Metz, KIBA, CASF-2016, DUD-E and BindingDB, to evaluate the effectiveness of our approach.

## INTRODUCTION

Compound–protein interactions (CPIs) play a critical role in drug discovery. To understand and quantify CPI, researchers traditionally employ biomedical measurement methods that focus on determining the inhibition constant ($K_{i}$), dissociation constant ($K_{d}$), half-maximal inhibitory concentration ($IC_{50}$) or half-maximal effective concentration ($EC_{50}$) values between drug candidates and target proteins, which rely on *in vitro* and *in vivo* experiments, and are trustworthy; however, they are associated with high costs and require significant time investment for development [1, 2].

Conventional virtual screening methods, such as docking-based methods, have been widely used because of their satisfactory performances. However, their prediction speed decreases significantly when the number of testing candidates is large, hindering their efficiency in handling massive datasets. Furthermore, as a prerequisite for accurate 3D information pertaining to both ligands and receptors, the efficacy of these methods significantly diminishes in instances where target-specific information is inadequately provided.

In contrast, the power of data-driven techniques on *in silico* dataset has revolutionized drug discovery for pharmaceutical companies. In recent decades, artificial intelligence (AI)-based methods, such as deep learning (DL) and machine learning, have gained considerable attention in various fields. Recognizing the strength of AI, many CPI prediction models have been constructed to use bio-cheminformatics datasets, then make predictions on the test pairs as binary decisions or continuum values following the primary task [3].

Two categories of models have demonstrated an outstanding ability to extract information from chemical compounds [4]. The first category includes deep neural networks, such as a multi-layer perceptron (MLP) neural network (DeepconvDTI [5]), and one-dimensional convolutional neural networks (1DCNN) (DeepDTA [6], HyperattentionDTI [7]) that work on descriptors or fingerprints. The second category comprises graph neural networks (GNNs) and their variants, which are used to gather insights from datasets with a graph-like structure (GraphDTA [8], TransformerCPI [9], PerceiverCPI [10]). Many previous studies have regarded the protein sequence as straightforward text and have employed a 1DCNN along with various techniques for protein sequence numbering. The compound and protein information is combined using concatenation or cross-attention techniques and then fed to the MLP layers to make predictions.

When considering the utilization of 3D information for CPI predictions such as Lim’s work [11], MINN-DTI [12] and Drug3D-DTI [13], it becomes evident that these endeavors are significantly reliant on pre-existing interaction pair structures. Notably, Lim’s work exclusively relies on the utilization of 3D datasets of existing molecular structures, while MINN-DTI is limited to extracting 3D information exclusively from proteins. Furthermore, Drug3D-DTI employs the use of RDKit to address the integration of 3D information derived from compounds. In regard to structure-based DL models like PIGNet [14], PLANET[15] and RTMScore [16], although these models have demonstrated commendable efficacy in predicting compound–protein interactions, their utility is contingent upon the ready availability of experimentally determined 3D protein structures. However, the experimental determination of protein structures can be time-consuming and expensive. This limitation restricts the number of proteins for which accurate structural information is available.

**Limitations**. The drawbacks of current methods are as follows:

1. Previous sequence-based studies represented protein sequences with plain text, limiting their ability to convey 3D conformation and extract atomic features.
2. Prior approaches have typically relied on pre-existing datasets to tackle the task at hand. Consequently, the voluminous dataset consisting of 3D information on compound conformers has not been incorporated.
3. The scarcity of comprehensive and well-structured datasets hampers the development, thereby impeding advancements in the accurate prediction and comprehensive understanding of CPI based on 3D information.
4. The prevalent practice of using the K-folds splitting method impedes the model’s capacity when confronted with substantially disparate test sets.

In this study, we address these challenges by proposing a DL-based approach called MulinforCPI (utilizing multi-level information for compound–protein interaction prediction). In the pre-training phase, we adopted a suggestion from 3DInfoMax to enable the GNN to generate 3D features from compounds [17]. During the fine-tuning phase, we generated the protein’s 3D fold representation from a single sequence using Evolutionary Scale Modeling Fold (ESMFold) [18], which was carried through multiple neural networks.

## METHODOLOGY

Due to the scarcity of datasets of sufficient size, particularly in the bioinformatics field, where reliable data are obtained from wet laboratories, we propose a novel approach using a transfer learning technique that takes advantage of knowledge from a pre-trained model.

**Figure 1:**
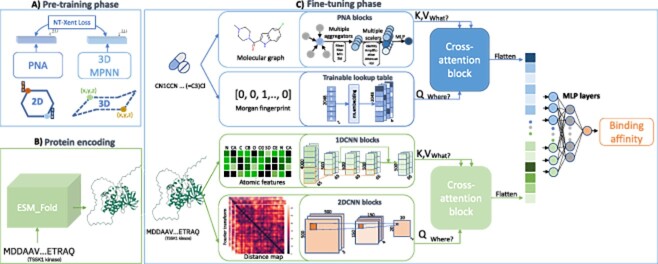
The schematic workflow of MulinforCPI encompasses a pipeline that primarily consists of three distinct components: (A) pre-training phase, which enables the PNA graph network to generate 3D features proficiently, (B) protein encoding, the protein sequence is encoded into a 3D structure and (C) fine-tuning phase, aimed at predicting CPIs.

In summary, the main objective of the proposed methodology is to gather information to address two inquiries: *‘where’* and *‘what’* to learn (as shown in Figure 1). The former relates to high-level information such as location information, which includes the presence of substructures of the molecule, or a distance map. In contrast, the latter concerns local information, focusing on capturing information from specific regions or localized areas, namely atomic features.

### Pre-training phase

#### Contrastive learning for 3D information compound encoding

Although 3D molecular knowledge is indispensable in representing compound properties, it is unachievable to procure 3D configurations at the magnitude necessary. To overcome this issue, we follow the suggestion of the training strategy proposed by [17] named 3Dinfomax, where the Quantum-Mechanical Properties of Drug-like Molecules (QMugs) dataset [19] is used for pre-training purposes, resulting in a GNN that is aware of 3D geometry information. The final goal of 3Dinfomax is to minimize the normalized temperature-scaled cross-entropy loss function 1 to maximize the similarity of positive pairs $z^{2D}_{i}$ and $z^{3D}_{i,j}$ when they come from the same molecule (same index $i$) and enforce dissimilarity between negative pairs ($z^{2D}_{i}$ and $z^{3D}_{k,j}$ where different index $i\neq k $): 


(1) \begin{align*}& \mathbf{L}_{NT-Xent} = -\frac{1}{N}\sum_{i=1}^{N}\left [log\frac{\sum_{j=1}^{c}e^{sim(z_{i}^{2D},z_{i,j}^{3D})/\tau }}{\sum_{_{k\neq i}^{k=1}}^{N}\sum_{j=1}^{c}e^{sim(z_{i}^{2D},z_{k,j}^{3D})/\tau}}\right ],\end{align*}

where $z^{2D}$ and $z^{3D}$ represent the outputs of the principal neighborhood aggregation (PNA) GNN [20] and message passing neural network with iteratively encoding the 3D coordinate information into the node features, respectively. $\tau $ denotes the temperature parameter, $c$ denotes the conformers and $N$ is the molecular graph.

We employed PNA for molecular geometry analysis with pre-training to overcome aggregation layer limitations and capture information from nearby nodes effectively. To combat over-smoothing, we used multiple aggregators, including mean, max, min and standard deviation. Our study also incorporated various techniques to enhance molecular representation, involving three scalers (identity, amplification and attenuation) and four readout aggregators (min, max, mean and sum).

### Fine-tuning phase

#### Compound encoding

After obtaining a 3D-aware PNA model, we have access to the GNN that can effectively tackle the question of *‘what’* to learn from the molecular graphical structure $G_{(V,E)}$. $V$ stands for atomic-level features, such as the chemical properties of each atom (e.g. electronegativity and hybridization), and bond-level features $E$, such as the bond type, which enables the model to capture the local features of the molecule. Subsequently, the output of PNA is represented by $O_{what}$.

In addition, to augment the capacity of the model to incorporate high-level information on the molecular structure, we employed Morgan fingerprints (MFs), commonly called circular fingerprints [21]. Using a binary vector, these fingerprints signify the presence of substructures within a particular radius. Through learning the concise representation of the molecular structure of a compound, our DL model can address the question of *‘where’* to learn.

In contrast to our previous work [10], in which we employed an MLP layer to extract patterns from the MFs, we adopted a learnable lookup table from the PyTorch library, specifically the nn.Embedding module, which was designed to learn embeddings of categorical variables. Our experiments revealed that nn.Embedding is more efficient and versatile for datasets with high sparsity, and the output of MFs networks can be shown as $O_{where}$.

**Figure 2:**
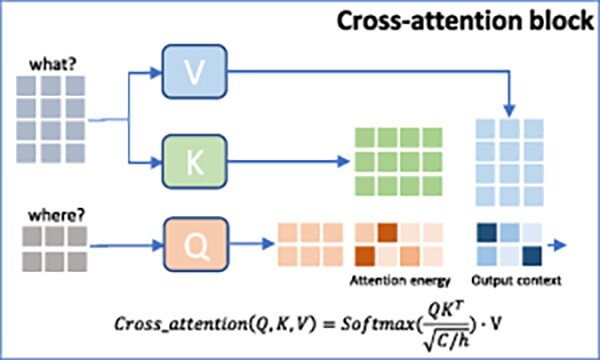
Cross-attention block where the attention mechanism enables the model to effectively capture information from multiple sources.

We employed a cross-attention technique to effectively incorporate local and high-level information from a compound, as shown in Figure 2. Here, the *‘what’* features are assigned the roles of Key and Value ($K, V$), while the *‘where’* features serve as the Query ($Q$). This arrangement is realized using three distinct projection functions ($f=\boldsymbol{\mathrm{w}}^{T}x+b$). Our approach draws significant inspiration from the capabilities of Perceiver IO proposed by [22] for efficiently handling diverse input modalities. The cross-attention mechanism is expressed as follows: 


(2) \begin{align*} & Q = f_{Q}(O_{where}); K=f_{K}(O_{what}); V=f_{V}(O_{what}); \end{align*}

(3) \begin{align*} & x_{comp} = CrossAttention(Q,K,V) = Softmax\left(\frac{QK^{T}}{\sqrt{C/h}} \right) \ast V, \end{align*}

where $C$ and $h$ are the embedding dimensions and the number of heads, respectively. Due to the inherent reliance of the real-world CPI prediction task on intricate chemical interactions, our intention is to enhance the representations of atomic features, denoted as *‘what’*, by incorporating supplementary contextual information, denoted as *‘where’*. Our objective is to improve the model’s comprehension of the intricate interplay between chemical information and the structural aspects of compounds.

#### Protein encoding

##### Protein preparation

Unlike previous sequence-based studies that treated protein sequences as plain text, we exploited the advanced DL model ESMFold, a fully end-to-end single-sequence structure predictor, to construct a 3D form of a protein from each protein sequence that is suitable for CPI prediction tasks. ESMFold uses unsupervised learning technique to train a family of transformer protein language model, ESM-2, on input sequences across diverse protein families. This architecture simplifies current state-of-the-art (SOTA) structure prediction models, avoiding the complex integration of multiple sequence alignment through attention mechanisms across rows and columns [23, 24].

The binding process relies solely on the complicated chemical attributes of atoms observed in proteins and ligands. Therefore, we can obtain atomic-level protein structures from primary sequences rather than just amino acid characteristics by implementing the predictions from ESMFold, which contains valuable information for DL.

##### Protein representation

After generating a 3D fold representation from the protein sequence, we could extract information from atomic features at the one-dimensional (1D) sequence level and 3D information encoded at the two-dimensional (2D) distance map level [25].

We first utilized the information that disclosed the atomic properties, including the specific type of atom in a given residue, the corresponding amino acid and the chemical element of the atom in the atomic-resolution structure of the protein. A one-hot encoding method was applied to handle categorical data representing an atom, and the results were concatenated. Consequently, a matrix $\mathbf{A}$=$(a_{i,j})_{1 \leq i\leq M,1 \leq j \leq N}$ is generated, where $M$ is the total number of atoms observed in the protein and $N$ is the concatenation of one-hot features. This information provides *‘what’* to focus on. To accomplish this, we employed 1DCNNs because they efficiently identify patterns from lengthy sequential datasets. 1DCNNs can learn to detect local patterns at various scales and over prolonged time windows by applying convolutional filters to input sequences.

Second, we extracted the residue-residue Euclidean distance information from the interatomic alpha carbon ($\alpha $-carbon or C$\alpha $) coordinates. The distance maps can reveal potential binding sites and interactions between different parts of the protein, which are useful for CPI predictions. Given two C$\alpha $ positions $i$($x_{1},x_{2},x_{3}$) and $j$($y_{1},y_{2},y_{3}$). We measured the distance by applying Equation: $d_{i,j} = \sqrt [2]{\sum _{c=1}^{3} (x_{c}- y_{c})^{2}}$, resulting in a pairwise distance feature matrix, $\mathbf{D}$=$(d_{i,j})_{1 \leq i,j \leq L}$, where $L$ is the length of the protein sequence. We chose to represent each amino acid residue using the $\alpha $-carbon because of its importance in protein folding.

When working with the matrix **D**, it is essential to consider the spatial relationships between the distance elements $d_{i,j}$. To effectively capture meaningful patterns, we perform one more step to map the distance matrix to a higher dimensional space using sine and cosine functions with high frequencies by a simple yet effective Fourier feature mapping function 4, which is theoretically motivated by [26] and empirically motivated by [17, 27]. Using sine and cosine functions, which introduce nonlinearity, more complex relationships that cannot be effectively represented in lower dimensional spaces can be captured. 


(4) \begin{align*}& \gamma (d_{i,j}) = \left[d_{i,j},\frac{sin(d_{i,j})}{2^{0}},\frac{cos(d_{i,j})}{2^{0}},...,\frac{cos(d_{i,j})}{2^{F-1}}\right]\end{align*}

Learning features from distance maps is essential because the arrangement of amino acids in a protein plays a critical role in its function. Two popular neural networks are available: transformers and CNNs. CNNs are preferred for their simplicity and computational efficiency, particularly for large images. Additionally, the transformer architecture requires a large dataset to converge, making CNNs a more practical choice for small dataset tasks, such as binding affinity prediction tasks. Moreover, we conducted a comparative analysis between MulinforCPI and an alternative model (where a transformer architecture replaced the CNN blocks), as depicted in Supplementary Tables 16 and 17. By acquiring features from the distance map, which provides high-level knowledge of the protein, our model can determine *‘where’* to learn from the protein.

To integrate the local and high-level features of the protein, we adopt a cross-attention technique where the atomic-level features are assigned key and value roles ($K,V$). In contrast, the residue-level features act as queries ($Q$). 


(5) \begin{align*} & Q = f_{Q}(T_{where}); K=f_{K}(T_{what}); V=f_{V}(T_{what}); \end{align*}

(6) \begin{align*} & x_{prot} = CrossAttention(Q,K,V), \end{align*}

where $T_{what}$ and $T_{where}$ represent the final outputs of 1DCNNs and 2DCNNs, respectively.

Cross-attention allows the model to capture complex relationships between atomic- and residue-level features by attending to relevant information. This attention mechanism enables the model to focus selectively on important features and discard irrelevant or noisy information.

In conclusion, a comprehensive pattern from multiple perspectives is essential for our model to gain a more profound insight into the atomic-level structure and features of a protein. This is achieved by taking information from *‘where’* and *‘what’* to learn given a protein.

#### Interaction

Finally, having obtained two final outputs to represent compounds and proteins, we decided to adopt a simpler method, concatenation, due to the complexity of the model. In the PerceiverCPI model [10], the final cross-attention block captures the altered information resulting from the interaction between the compound and protein. However, we experimentally observed that the cross-attention technique employed in a previous study exhibited suboptimal performance when applied to highly sparse datasets. In addition, this technique yielded results comparable with those achieved using the MulinforCPI method on the Davis dataset. Consequently, we transfer these outputs to two MLP layers to enhance the precision of the predictions. 


(7) \begin{align*}& \begin{matrix} z = \sigma (\mathbf{w}_{z}^{T}(x_{comp},x_{prot})+b_{z})\\ \\ \hat{y}= \sigma (\mathbf{w}_{o}^{T}(z)+b_{o}), \end{matrix}\end{align*}

where $x_{comp}$ and $x_{prot}$ denote the final outputs of the two networks and $\sigma $ represents the activation function.

## DATASETS

In the pre-training task, the QMugs compilation encompasses quantum mechanical features of over 665 000 molecules with significant biological and pharmacological importance derived from the ChEMBL database. This corresponds to approximately 2 million conformers, as indicated in Table 1. The conformers of a compound refer to the different spatial arrangements or configurations that the molecule can adopt while maintaining the same connectivity between atoms. These conformations arise from the rotation of the single bonds in the molecule. Each conformer represents a distinct arrangement of atoms in three-dimensional space.

**Table 1:** Descriptive statistics of QMugs dataset.

Dataset	Unique compounds	Total conformations	Heavy atoms max (mean)
QMugs	665 911	1992 984	100 (30.6)

We evaluated our models against SOTA models for the regression task using four well-established datasets, as shown in Table 2. Because of the 100 percent density of the Davis dataset, which covers approximately 80% of the human catalytic protein kinomes, we conducted three experiments: novel pairs, novel compounds and novel protein settings. Only a novel hard pair setting was used for the KIBA and Metz datasets.

**Table 2:** Statistics of the benchmark datasets.

Dataset	Task	Proteins	Drugs	Interactions		Density (%)
				Negatives	Positives	
Davis	Regression	442	68	30 056		100
KIBA	Regression	229	2068	117 657		24,84
Metz	Regression	170	1423	35 259		14,57
CASF-2016	Regression	15	57	57		6.6
DUD-E Diverse	Classification	7	108 212	107 590	1759	14,43
BindingDB	Classification	813	49 752	27 493	33 777	0,15

We conducted an experiment involving zero-shot testing on a subset of the CASF-2016 benchmark dataset. The choice of this dataset was justified by its reputation as a benchmark for comparing different docking scoring methods. We filtered to select ligands within the weight range of 300 (Dalton) to 650 (Dalton). Our objective was to obtain ligands with molecular weights comparable with those of small-molecule drugs. Within the processed dataset, it has been ascertained that each protein is associated with a minimum of four distinct drugs capable of binding to it. In addition, the $p_{Ki}$-binding affinities ranged from 2.4 to 11.15. Moreover, this dataset lends itself well to docking simulations.

**Figure 3:**
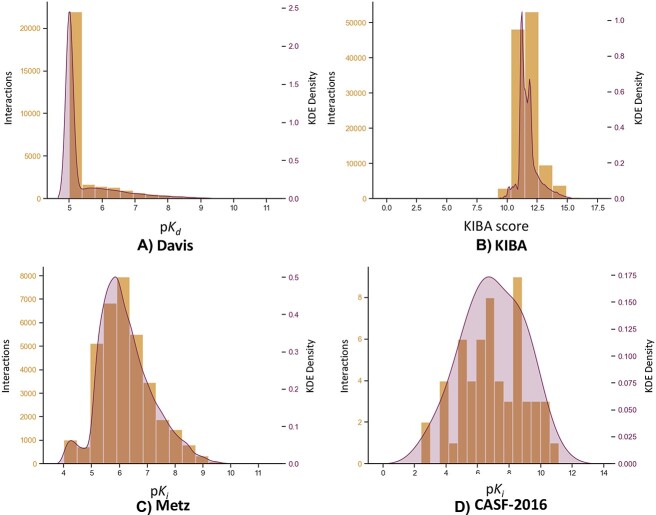
The label histogram and label density estimation of four regression datasets. (A) Davis dataset, (B) KIBA dataset, (C) Metz dataset and (D) CASF-2016 dataset.

The results in Figure 3 illustrate the label distributions of four benchmark datasets. The Davis and KIBA datasets exhibited skewed distributions, whereas the Metz dataset and CASF-2016 dataset exhibited well-distributed labels. This disparity in label distribution contributed to more effective learning outcomes.

**Figure 4:**
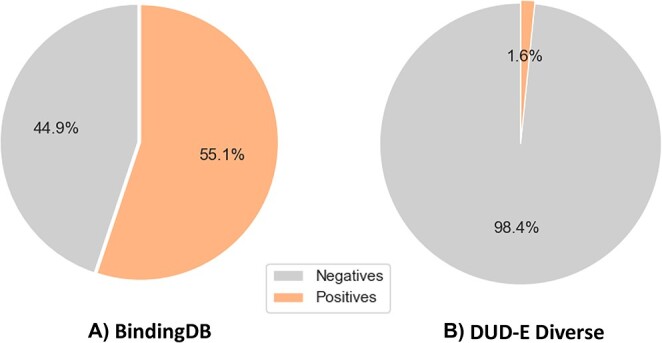
The pie chart of two classification datasets. (A) BindingDB dataset, (B) DUD-E Diverse dataset.

Furthermore, compared with the classification models, we reconstituted the training dataset from the BindingDB dataset for the enrichment factor task [28]. Conforming to the discourse on the activity threshold discussed in the literature, we labeled the interactions as positive if their $IC_{50}$ value was less than 100 nM and negative if their $IC_{50}$ value exceeded 10 000 nM. Figure 4 provides an insightful visualization of the relative proportions of positive and negative instances within two distinct datasets.

## CROSS-CLUSTER VALIDATION

Instead of using the conventional k-fold separation method, commonly used in previous studies, we adopted a cluster cross-validation technique. This technique is an advancement in the similarity-split cross-validation method. This method guarantees that compounds within the same cluster do not end up in the training or testing sets, as it applies to proteins.

**Figure 5:**
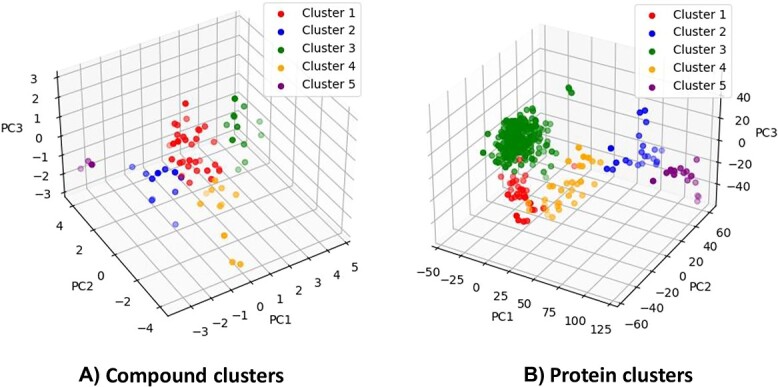
Demonstration of cluster cross-validation for DAVIS dataset by principal component analysis. (A) compound clusters and (B) protein clusters.

In our approach, we group compounds using the Butina clustering algorithm by [29]. This algorithm is hierarchical and relies on Tanimoto similarity coefficients for compound clustering, computed through pairwise comparisons. These coefficients were computed using the molecular fingerprints generated by the RdKit library. For the clustering of proteins, we used the k-means clustering method, which involves grouping a given set of data points into K clusters based on their Euclidean distance metrics. Figure 5 illustrates the clusters for proteins and compounds in the Davis dataset. Unlike the separation approach proposed by [30], our methodology employs a hierarchical algorithm for clustering compounds because of its effectiveness in identifying structurally similar molecules, which leads to more precise and constrained cluster formation. Tables 9 and 13 in the Supplementary demonstrate the validity of our technique in effectively reducing the similarity between the training and test interactions.

The proposed separation strategy enhances the generality of the model in real-world applications by creating a clear distinction between the training and test sets such that they exhibit significant dissimilarities.

## EXPERIMENTS

### Experimental settings

To compare the performance of the proposed method with SOTA models, we used the following five settings:

- Novel pair (Davis): No overlaps exist between the training and test datasets. Neither the training compound nor the training protein appeared in the test set.
- Novel compound (Davis): No intersections of compounds exist in the training set and compounds in the test set.
- Novel protein (Davis): No intersections of proteins exist in the training set and proteins in the test set.
- Novel hard pair (Metz, KIBA): No overlaps exist between the training and test datasets. For the testing interactions, we specifically selected those with similarities below 0.3 (We removed interactions from the training dataset if either the protein sequence or the compound had a similarity score exceeding the threshold).
- Cross-domain (Metz, CASF-2016): No overlaps exist in interactions between the training set and the test set. We removed interactions involving 56 proteins and 105 compounds with similarities higher than 0.3 from the Metz dataset.
- Enrichment factor analysis (BingdingDB, diverse DUD-E): No overlaps of interactions exist between the training set (BingdingDB) and diverse test set (we removed interactions for two proteins and compounds that appeared in both datasets (GCR_HUMAN (P04150) and AKT1_HUMAN (P31749) and 102 compounds) from the training set).

### Metrics

We assessed the compatibility between MulinforCPI and its competitors using three main metrics: mean squared error (MSE), concordance index (C-index) and Spearman correlation coefficient ($\rho $). 


(8) \begin{align*} MSE = \frac{1}{n}\ast \sum_{i=0}^{n}(y_{i}-\hat{y}_{i})^{2} \end{align*}

(9) \begin{align*} \begin{split} C\hbox{-}Index = \frac{\sum_{i,j}1_{y_{i}>y_{j}}\cdot 1_{\hat{y}_{i}>\hat{y}_{j}}}{\sum_{i,j}1_{y_{i}>y_{j}}} \\ with: \quad 1_{y_{i}<y_{j}}=1 \quad if \quad y_{i}<=y_{j} \quad else\quad 0 \\ 1_{\hat{y}_{i}<\hat{y}_{j}}=1 \quad if \quad \hat{y}_{i}<=\hat{y}_{j} \quad else\quad 0 \end{split} \end{align*}

(10) \begin{align*} \rho = 1-\frac{6\sum_{i=1}^{i=n} d^{2}_{i}}{n(n^{2}-1)}, \end{align*}

where ${y}$ is the ground truth value, $\hat{y}$ is the corresponding prediction, $d$ is the sum of the squared differences between the ranks of the corresponding pairs of values in the ${y}$ and $\hat{y}$ arrays and $n$ is the number of predictions.

$\rho $ provides information regarding the strength and direction of the monotonic relationship between two variables. In contrast, MSE measures the average squared difference between the predicted and actual values of the dependent variable. Therefore, they can be used to evaluate the predictive accuracy of the models. The C-index metric is useful in survival analysis to estimate confidence intervals around model performance measures.

Furthermore, we used enrichment factors at 1 percent (EF$_{1\%}$) and Boltzmann-Enhanced Discrimination of Receiver Operating Characteristic with a specific parameter value of 80.5 (BEDROC$_{\alpha =80.5}$) to show the performance of all models in decoy classification experiments. EF$_{1\%}$ refers to the enrichment of true-positive interactions within the top 1 percent of the predictions. In addition, BEDROC$_{\alpha =80.5}$ was calculated based on the area under the interpolated precision-recall curve. We used the alpha value recommended by [31].

### Experimental results

To assess the predictive capability of our proposed approach, we conducted a comparative analysis with SOTA end-to-end DL methodologies and docking-based programs. The outcomes of the four experiments, namely novel pair, novel compound, novel protein and novel hard pair, were obtained using a 5-fold cluster cross-validation technique. Meanwhile, the result of the cross-domain experiment was acquired by zero-shot testing. We comprehensively evaluated MulinforCPI alongside SOTA competitors for two fundamental tasks: regression and classification. We use the binary cross-entropy loss and the MSE loss for classification and regression, respectively.

The primary objective of this study was to examine the performance of various SOTA models in three novel settings using regression datasets. However, all these models exhibited low $\rho $ values in novel pair settings, indicating their limited capacity to predict the target based on the learned features. Nevertheless, the MulinforCPI model showed robustness in learning from the training datasets, consistently achieving the highest CI values and lowest MSE across most experiments. More specifically, high CI suggests that the model has a strong ability to predict outcomes, which is generally desirable in predictive modeling. In the novel pair settings across the three benchmark datasets, the proposed method, MulinforCPI, attained the lowest MSE, thereby indicating its capability to generate predictions that closely align with real labels compared with the baseline models as shown in Table 3.

**Table 3:** Restult for novel-pair in Davis dataset (MSE $\downarrow $ better, CI $\uparrow $ better, Spearman Correlation $\uparrow $ better, mean and standard deviation values were computed from 5-fold results’ averages).

Models	MSE	CI	Spearman Correlation
DeepDTA	0.719($\pm $0.312)	0.456($\pm $0.107)	−0.054($\pm $0.162)
DeepConvDTI	0.602($\pm $0.221)	0.580($\pm $0.065)	0.141($\pm $0.105)
TransformerCPI	0.565($\pm $0.252)	0.552($\pm $0.024)	0.087($\pm $0.037)
GraphDTA (GINs)	1.078($\pm $0.564)	0.499($\pm $0.100)	0.011($\pm $0.139)
HyperattentionDTI	0.633($\pm $0.249)	0.529($\pm $0.046)	0.049($\pm $0.078)
PerceiverCPI	0.668($\pm $0.357)	0.547($\pm $0.071)	0.062($\pm $0.124)
MulinforCPI (ours)	**0.547($\pm $0.256)**	**0.646($\pm $0.05)**	**0.237($\pm $0.061)**
MulinforCPI (ours) Freeze 95%	0.580($\pm $0.258)	0.528($\pm $0.073	0.055($\pm $0.093)

Our experimental results revealed that the models trained on datasets characterized by well-distributed labels, such as the Metz dataset, exhibited superior predictive performance. These models yielded higher Spearman correlation coefficients than those trained on datasets with skewed label distributions, such as the Davis and KIBA datasets, which can be seen in the Supplementary material (Table 2). These results indicate that the models can generate more accurate predictions for unseen test sets where the model has no prior information regarding the test interactions. Our analysis of the Metz dataset includes two experiments: a cross-domain experiment and a novel pair setting. We observe a moderate correlation in both cases, as seen in Table 6 and Supplementary Table 3 (To enhance the comprehensibility of our work, we have relocated the results pertaining to the Metz and KIBA datasets to the supplementary document).

The unfreeze–freeze technique revolutionizes transfer learning for better performance in new domains, offering benefits like reduced computational requirements and improved generalization. In our experiments, we froze the upper layers of the PNA network to prevent it from being updated during subsequent training, thus ensuring that the model’s previously acquired knowledge remained intact. We empirically set the freezing threshold based on the depth of the PNA network in MulinforCPI *[0, 0.95]*. Tables 4 and 5 demonstrate that reducing the number of learnable parameters in the model leads to improved prediction capability. However, when novel pair settings are considered, the effectiveness of the techniques is diminished, primarily because of the limited size of the dataset. In scenarios where the dataset is smaller or less diverse, freezing and unfreezing layers can hinder the ability of the model to learn and generalize effectively.

**Table 4:** Result for novel-comp in Davis dataset (MSE $\downarrow $ better, CI $\uparrow $ better, Spearman Correlation $\uparrow $ better, mean and standard deviation values were computed from 5-fold results’ averages).

Models	MSE	CI	Spearman correlation
DeepDTA	0.873($\pm $0.274)	0.549($\pm $0.036)	0.086($\pm $0.068)
DeepConvDTI	0.750($\pm $0.275)	0.674($\pm $0.048)	0.312($\pm $0.075)
TransformerCPI	0.831($\pm $0.244)	0.615($\pm $0.039)	0.205($\pm $0.051)
GraphDTA (GINs)	0.750($\pm $0.283)	0.688($\pm $0.05)	**0.333($\pm $0.062)**
HyperattentionDTI	0.757($\pm $0.269)	0.589($\pm $0.057)	0.157($\pm $0.104)
PerceiverCPI	0.746($\pm $0.245)	0.669($\pm $0.036)	0.303($\pm $0.054)
MulinforCPI (ours)	0.690($\pm $0.275)	0.679($\pm $0.072)	0.317($\pm $0.113)
MulinforCPI (ours) Freeze 95%	**0.679($\pm $0.219)**	**0.688($\pm $0.028)**	0.290($\pm $0.084)

**Table 5:** Result for novel-prot in Davis dataset (MSE $\downarrow $ better, CI $\uparrow $ better, Spearman Correlation $\uparrow $ better, mean and standard deviation values were computed from 5-fold results’ averages).

Models	MSE	CI	Spearman correlation
DeepDTA	0.529($\pm $0.130)	0.729($\pm $0.014)	0.396($\pm $0.031)
DeepConvDTI	**0.465($\pm $0.151)**	0.755($\pm $0.062)	0.433($\pm $0.094)
TransformerCPI	0.487($\pm $0.172)	0.660($\pm $0.040)	0.278($\pm $0.066)
GraphDTA (GINs)	1.122($\pm $0.887)	0.694($\pm $0.051)	0.333($\pm $0.088)
HyperattentionDTI	0.542($\pm $0.219)	0.707($\pm $0.040)	0.352($\pm $0.044)
PerceiverCPI	0.513($\pm $0.213)	0.748($\pm $0.022)	0.427($\pm $0.033)
MulinforCPI (ours)	0.488($\pm $0.138)	**0.756($\pm $0.017)**	**0.439($\pm $0.022)**
MulinforCPI (ours) Freeze 95%	0.478($\pm $0.140)	0.753($\pm $0.020)	0.435($\pm $0.027)

In the cross-domain experiment, we made a comparison with three well-known docking simulations: Glide [32], Autodock-GPU (AutoDock version 4.2.6) [33] and Autodock-Vina (version 1.2.3) [34]. As shown in Table 6, none of the data-driven methods matched the performance of the first-principles methods. Despite MulinforCPI outperforming its DL competitors in this task, our approach failed to achieve the robust correlation exhibited by Glide. Docking simulations involve generating potential ligand positions and orientations within the binding site, then evaluating each pose using a scoring function. The goal is to systematically explore the ligand/receptor’s conformational space to find the best binding position with the lowest energy. In contrast, DL methods often need abundant labeled data for training, which can be challenging to obtain in CPI domains or resource-intensive to create.

**Table 6:** The results cross-domain experiments when similarity threshold = 0.3 (MSE $\downarrow $ better, CI $\uparrow $ better, Spearman Correlation $\uparrow $ better).

Model	MSE	CI	Spearman correlation
DeepDTA	6.193	0.542	0.135
DeepConvDTI	6.611	0.562	0.176
TransformerCPI	4.999	0.6	0.298
GraphDTA (GINs)	6.676	0.512	0.02
HyperattentionDTI	5.484	0.606	0.314
PerceiverCPI	5.279	0.615	0.342
MulinforCPI (ours)	4.698	0.602	0.297
MulinforCPI (ours) Freeze 95%	**4.391**	0.642	0.395
Autodock-GPU	N/A	0.717	**0.620**
Autodock-Vina	N/A	0.711	0.608
Glide	N/A	**0.722**	0.614

We compared our method with five well-known first-principles methods (Gold [35], Surflex [36], FlexX [37], Blaster [38] and Glide) on an enrichment factor analysis task. This task quantitatively measures a model’s performance in retrieving true-positive interactions from a large pool of candidates and helps in benchmarking and comparing different models in drug–target interaction prediction tasks. For enhanced clarity, we describe the results obtained by random estimation. This entailed making arbitrary predictions regarding the probability of binding, ranging from 0 to 1, regardless of the information from the interaction pair (the results were obtained by computing the average of three independent iterations of the guessing experiments) as shown in Table 7. Our findings indicated that the MulinforCPI model could identify true-positive pairs across all targets. Because of the considerable number of interactions within the test set derived from the DUD-E dataset, we experienced difficulties executing the experiments with Autodock-GPU and Autodock-Vina. Nevertheless, we relied on the information in the original paper for the qualitative results.

**Table 7:** The enrichment factor analysis results on a Diverse subset from the DUD-E database (EF$_{1\%}$$\uparrow $ better, BEDROC$_{\alpha =80.5}$$\uparrow $ better, mean and standard deviation values were computed from per protein results’ averages).

Models	EF$_{1\%}$ ($\pm $std)	BEDROC$_{\alpha =80.5}$ ($\pm $std)
DeepConvDTI	6.357($\pm $6.173)	0.118($\pm $0.109)
TransformerCPI	7.039($\pm $12.496)	0.117($\pm $0.192)
HyperattentionDTI	1.753($\pm $2.551)	0.038($\pm $0.051)
PerceiverCPI	4.649($\pm $3.136)	0.094($\pm $0.067)
MulinforCPI (ours)	7.886($\pm $10.642)	0.137($\pm $0.167)
MulinforCPI (ours) Freeze 95%	4.248($\pm $5.787)	0.078($\pm $0.095)
Random Guessing	0.940($\pm $0.844)	0.022($\pm $0.010)
Gold	N/A	0.253($\pm $0.182)
Glide	N/A	**0.259($\pm $0.171)**
Surflex	N/A	0.119($\pm $0.093)
FlexX	N/A	0.104($\pm $0.060)
Blaster	**13.571($\pm $12.908)**	N/A

## DISCUSSION

In our experiments, we observed that none of the SOTA models that used the protein sequence as plain text was successful in accurately predicting the interaction in all settings. Based on the $\rho $ coefficient, this indicates an inability to demonstrate satisfactory performance on the test set. This inadequate performance can be attributed to the limited availability of curated datasets designed explicitly for CPI prediction tasks. Nonetheless, we believe that with the rapid accumulation of datasets, there is potential for the gap between data-driven and first-principles methods to diminish over time. Moreover, our approach to incorporating 3D information and transfer learning techniques demonstrated superior performance compared with previous canonical approaches. By leveraging multi-resolution techniques, we identified a suitable direction for the long-term advancement of this task.

**Figure 6:**
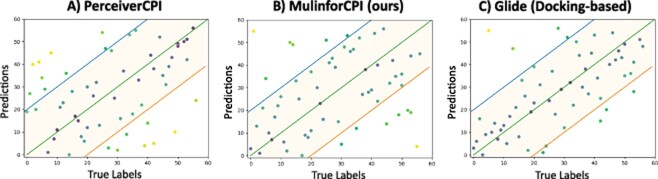
The scatter plot visualization of ranking predictions between data-driven methods (A,B) and a docking-based method (C) in the cross-domain experiment.

We visualized the ranking predictions from three typical models, including the first-principle and data-driven methods, regarding the prediction rankings, as shown in Figure 6 (The full visualization can be found at Supplementary Figure 1). This figure demonstrates that a significant portion of the ranking predictions generated by the various data-driven methods for the subset from CASF-2016 were arbitrary. Conversely, predictions derived from MulinforCPI and first-principles methods exhibit superior performance, exhibiting a pronounced linear relationship between the predicted and actual rankings. In three specific examples, MulinforCPI accurately predicted the ranks of the testing points. The intensity of the colors indicates the accuracy of the predictions, with lighter shades representing poorer predictions and darker shades indicating more accurate predictions.

DL models have the potential to function as valuable filters, thereby significantly expediting drug discovery. A high-speed inference runtime is essential for tasks such as high-throughput virtual screening of drug candidates and reverse screening to identify protein targets [39]. By combining the strengths of data-driven methods with those of first-principles methods, an efficient and robust approach can be established [40].

## CONCLUSION AND FUTURE WORK

### Conclusion

In this study, we developed a DL framework that leverages multi-level information from both the compound and protein of the interaction by adopting the transfer learning technique. Instead of conducting end-to-end training of deep neural networks based solely on binding data, we opt for pre-training the embeddings for compounds using a more extensive chemical feature space. This approach, combined with the protein fold predictions, enabled us to extend the scope of the CPI prediction task to encompass chemical structures beyond those encountered in the training data. Furthermore, we have also proposed a splitting method that helps researchers avoid the potential overlap between training and test sets.

### Future work

Notwithstanding that the outstanding performance of the proposed network, considerable work is required to enhance the performance of CPI prediction tasks in the future.

- Based on the data obtained from ESMFold, MulinforCPI requires a substantial amount of memory for preprocessing before proceeding to GPU training. Enhancing the input while maintaining optimal performance can accelerate the training process.
- The interpretability of our DL network is constrained by the dimensionality reduction of the CNNs and the MLP layers. Addressing these significant characteristics will form an integral part of future endeavors.
- Leveraging equivariant networks, such as E(n) Equivariant GNNs [41] and Euclidean Neural Networks [42], to incorporate positional information (rotation, translation, inversion) has the potential to enhance the model’s capacity to capture more informative patterns.

**Key Points**

- We propose that the MulinforCPI DL model, which utilizes multi-level information from compounds and proteins, can address significant challenges in CPI prediction tasks.
- In contrast to prior research where most end-to-end models used sequences of amino acid characters to conduct protein representations, our approach involved leveraging both atomic-level attributes and 3D information extracted from proteins to augment the model’s capacity.
- The developed transfer learning technique leverages the extensive Quantum-Mechanical Properties of Drug-like Molecules (QMugs) dataset and employs it for fine-tuning of CPI datasets.
- Our separation strategy enables the model to closely approximate the actual problem when faced with unfamiliar test sets.
- Our research reveals the gap between first-principle methods and data-driven approaches. We believe these findings open up opportunities for future research on CPI prediction tasks.

## Supplementary Material

MulinforCPI_sup_1st_rev_bbad484

## Data Availability

The source data and codes of our network and related links for experimental datasets are available on GitHub at https://github.com/dmis-lab/MulinforCPI.

## Author Biographies

**Ngoc-Quang Nguyen** is a PhD student at Korea University, focuses on multimodal learning, 3D geometric information extraction, and precision medicine. His research integrates data science, artificial intelligence, machine learning, and formal reasoning to accelerate bio/medical science.

**Sejeong Park** is a PhD student at Korea University, where she applies deep learning techniques to cheminformatics to advance new drug development. Her work includes developing drug discovery pipelines utilizing various molecular simulation tools. Additionally, Park is currently serving as a Senior Scientist at AIGEN Sciences, where she performs a range of important roles in her field.

**Mogan Gim** is a postdoctoral researcher at Korea University, South Korea, where he also received his PhD. His primary research interests revolve application of cheminformatics to AI-driven precision nutrition and drug discovery. His research work also encompasses other domain-specific AI-driven applications such as cooking knowledge, portfolio optimization, legal reasoning and bioinformatics as well.

**Jaewoo Kang** is a Professor of Computer Science and Engineering at Korea University and the CEO and Founder of AIGEN Sciences Inc. His primary research interest lies in developing artificial intelligence to address unmet needs in biomedicine.

## References

[1] Bai P , Miljković F, John B, Haiping L. Interpretable bilinear attention network with domain adaptation improves drug–target prediction. Nature Mach Intell 2023;1–11.

[2] Wan X , Xiaolong W, Wang D, et al. An inductive graph neural network model for compound–protein interaction prediction based on a homogeneous graph. Brief Bioinform 2022;23. 10.1093/bib/bbac073 PMC9310259 35275993

[3] Zhang R , Wang Z, Wang X, et al. Mhtan-dti: Metapath-based hierarchical transformer and attention network for drug–target interaction prediction. Brief Bioinform 2023;24(2):bbad079. 10.1093/bib/bbad079 36892155

[4] Yang K , Swanson K, Jin W, et al. Analyzing learned molecular representations for property prediction. J Chem Inf Model 2019;59(8):3370–88. 31361484 10.1021/acs.jcim.9b00237 PMC6727618

[5] Lee I , Keum J, Nam H. Deepconv-dti: prediction of drug-target interactions via deep learning with convolution on protein sequences. PLoS Comput Biol 2019;15(6):e1007129. 10.1371/journal.pcbi.1007129 PMC6594651 31199797

[6] Öztürk H , Özgür A, Ozkirimli E. Deepdta: deep drug–target binding affinity prediction. Bioinformatics 2018;34(17):i821–9. 30423097 10.1093/bioinformatics/bty593 PMC6129291

[7] Zhao Q , Zhao H, Zheng K, Wang J. Hyperattentiondti: improving drug–protein interaction prediction by sequence-based deep learning with attention mechanism. Bioinformatics 2022;38(3):655–62. 34664614 10.1093/bioinformatics/btab715

[8] Nguyen T , Le H, Quinn TP, et al. Graphdta: predicting drug–target binding affinity with graph neural networks. Bioinformatics 2021;37(8):1140–7. 33119053 10.1093/bioinformatics/btaa921

[9] Chen L , Tan X, Wang D, et al. Transformercpi: improving compound–protein interaction prediction by sequence-based deep learning with self-attention mechanism and label reversal experiments. Bioinformatics 2020;36(16):4406–14. 32428219 10.1093/bioinformatics/btaa524

[10] Nguyen N-Q , Jang G, Kim H, Kang J. Perceiver cpi: a nested cross-attention network for compound–protein interaction prediction. Bioinformatics 2023;39(1):btac731. 36416124 10.1093/bioinformatics/btac731 PMC9848062

[11] Lim J , Ryu S, Park K, et al. Predicting drug–target interaction using a novel graph neural network with 3d structure-embedded graph representation. J Chem Inf Model 2019;59(9):3981–8. 31443612 10.1021/acs.jcim.9b00387

[12] Li F , Zhang Z, Guan J, Zhou S. Effective drug–target interaction prediction with mutual interaction neural network. Bioinformatics 2022;38(14):3582–9. 35652721 10.1093/bioinformatics/btac377 PMC9272808

[13] Liao Z , Huang X, Mamitsuka H, Zhu S. Drug3d-dti: improved drug-target interaction prediction by incorporating spatial information of small molecules. In:In 2021 IEEE International Conference on Bioinformatics and Biomedicine (BIBM). IEEE, 2021, 340–7.

[14] Moon S , Zhung W, Yang S, et al. Pignet: a physics-informed deep learning model toward generalized drug–target interaction predictions. Chem Sci 2022;13(13):3661–73. 35432900 10.1039/d1sc06946b PMC8966633

[15] Zhang X , Gao H, Wang H, et al. Planet: a multi-objective graph neural network model for protein–ligand binding affinity prediction. J Chem Inf Model 2023. 10.1021/acs.jcim.3c00253 37319418

[16] Shen C , Zhang X, Deng Y, et al. Boosting protein–ligand binding pose prediction and virtual screening based on residue–atom distance likelihood potential and graph transformer. J Med Chem 2022;65(15):10691–706. 35917397 10.1021/acs.jmedchem.2c00991

[17] Stärk H , Beaini D, Corso G, et al. 3d infomax improves gnns for molecular property prediction. In International Conference on Machine Learning, pages 20479–502. PMLR, 2022.

[18] Lin Z , Akin H, Rao R, et al. Evolutionary-scale prediction of atomic-level protein structure with a language model. Science 2023;379(6637):1123–30. 36927031 10.1126/science.ade2574

[19] Isert C , Atz K, Jiménez-Luna J, Schneider G. Qmugs, quantum mechanical properties of drug-like molecules. Scientific Data 2022;9(1):273. 35672335 10.1038/s41597-022-01390-7 PMC9174255

[20] Corso G , Cavalleri L, Beaini D, et al. Principal neighbourhood aggregation for graph nets. Adv Neural Inf Process Syst 2020;33:13260–71.

[21] Rogers D , Hahn M. Extended-connectivity fingerprints. J Chem Inf Model 2010;50(5):742–54. 20426451 10.1021/ci100050t

[22] Jaegle A , Borgeaud S, Jean-Baptiste Alayrac, et al. Perceiver io: A general architecture for structured inputs & outputs. In International Conference on Learning Representations, 2022.

[23] Rao RM , Liu J, Verkuil R, et al. Msa transformer. In International Conference on Machine Learning, pages 8844–56. PMLR, 2021.

[24] Jumper J , Evans R, Pritzel A, et al. Highly accurate protein structure prediction with alphafold. Nature 2021;596(7873):583–9. 34265844 10.1038/s41586-021-03819-2 PMC8371605

[25] Baek M , DiMaio F, Anishchenko I, et al. Accurate prediction of protein structures and interactions using a three-track neural network. Science 2021;373(6557):871–6. 34282049 10.1126/science.abj8754 PMC7612213

[26] Rahaman N , Baratin A, Arpit D, et al. On the spectral bias of neural networks. In International Conference on Machine Learning, pages 5301–10. PMLR, 2019.

[27] Tancik M , Srinivasan P, Mildenhall B, et al. Fourier features let networks learn high frequency functions in low dimensional domains. Adv Neural Inf Process Syst 2020;33:7537–47.

[28] Gilson MK , Liu T, Baitaluk M, et al. Bindingdb in 2015: a public database for medicinal chemistry, computational chemistry and systems pharmacology. Nucleic Acids Res 2016;44(D1):D1045–53. 26481362 10.1093/nar/gkv1072 PMC4702793

[29] Butina D . Unsupervised data base clustering based on daylight’s fingerprint and tanimoto similarity: a fast and automated way to cluster small and large data sets. J Chem Inf Comput Sci 1999;39(4):747–50.

[30] Mayr A , Klambauer G, Unterthiner T, et al. Large-scale comparison of machine learning methods for drug target prediction on chembl. Chem Sci 2018;9(24):5441–51. 30155234 10.1039/c8sc00148k PMC6011237

[31] Chaput L , Martinez-Sanz J, Saettel N, Mouawad L. Benchmark of four popular virtual screening programs: construction of the active/decoy dataset remains a major determinant of measured performance. J Chem 2016;8(1):1–17. 10.1186/s13321-016-0167-x PMC5066283 27803745

[32] Friesner RA , Banks JL, Murphy RB, et al. Glide: a new approach for rapid, accurate docking and scoring. 1. Method and assessment of docking accuracy. J Med Chem 2004;47(7):1739–49. 15027865 10.1021/jm0306430

[33] Santos-Martins D , Solis-Vasquez L, Tillack AF, et al. Accelerating autodock4 with gpus and gradient-based local search. J Chem Theory Comput 2021;17(2):1060–73. 33403848 10.1021/acs.jctc.0c01006 PMC8063785

[34] Trott O , Olson AJ. Autodock vina: improving the speed and accuracy of docking with a new scoring function, efficient optimization, and multithreading. J Comput Chem 2010;31(2):455–61. 19499576 10.1002/jcc.21334 PMC3041641

[35] Jones G , Willett P, Glen RC, et al. Development and validation of a genetic algorithm for flexible docking. J Mol Biol 1997;267(3):727–48. 9126849 10.1006/jmbi.1996.0897

[36] Jain AN . Surflex: fully automatic flexible molecular docking using a molecular similarity-based search engine. J Med Chem 2003;46(4):499–511. 12570372 10.1021/jm020406h

[37] Rarey M , Kramer B, Lengauer T, Klebe G. A fast flexible docking method using an incremental construction algorithm. J Mol Biol 1996;261(3):470–89. 8780787 10.1006/jmbi.1996.0477

[38] Irwin JJ , Shoichet BK, Mysinger MM, et al. Automated docking screens: a feasibility study. J Med Chem 2009;52(18):5712–20. 19719084 10.1021/jm9006966 PMC2745826

[39] Corso G , Stärk H, Jing B, et al. Diffdock: Diffusion steps, twists, and turns for molecular docking. In NeurIPS 2022 Workshop on Score-Based Methods.

[40] Stärk H , Ganea O, Pattanaik L, Barzilay R, Jaakkola T. Equibind: Geometric deep learning for drug binding structure prediction. In International Conference on Machine Learning, pages 20503–21. PMLR, 2022.

[41] Satorras VG , Hoogeboom E, Welling M. E (n) equivariant graph neural networks. In International conference on machine learning, pages 9323–32. PMLR, 2021.

[42] Geiger M , Smidt T. e3nn: Euclidean neural networks, 2022.

