# Supplementary material for: MulinforCPI: enhancing precision of compound–protein interaction prediction through novel perspectives on multi-level information integration
Source: Brief Bioinform. 2024 Jan 4;25(1):bbad484. doi: 10.1093/bib/bbad484 (PMC10768804; doi:10.1093/bib/bbad484)
Supplement: MulinforCPI_sup_1st_rev_bbad484 [file mulinforcpi_sup_1st_rev_bbad484.pdf]

Supplemental information for: "MulinforCPI: enhancing precision of compound-protein interaction prediction through novel perspectives on multi-level information integration"

Ngoc-Quang Nguyen[1], Sejeong Park[1,3], Mogan Gim[1], and Jaewoo Kang[1,2,3,*]

[1]Department of Computer Science and Engineering, Korea University, Seoul, Republic of Korea

[2]Interdisciplinary Graduate Program in Bioinformatics, Korea University, Seoul, Republic of Korea

[3]AIGEN Sciences, Seoul, 04778, Republic of Korea

[*]Corresponding author: kangj@korea.ac.kr

1 Ablation study

Ablation study is a crucial component of deep learning model evaluation and analysis. It involves systematically removing or disabling specific components or modules within a model to understand their individual contributions and assess their impact on overall performance. The main goal of an ablation study is to identify the most influential factors or components within a deep learning model and gain insights into how they contribute to the model's performance. By selectively removing or modifying these components and observing the resulting changes, we can assess their significance and understand their role in achieving the desired outcomes. In here, we selectively removed components from MulinforCPI.

Table 1: Ablation study of MulinforCPI.(MSE: Mean Squared Error, CI: Concordance Index, PC: Pearson Correlation, ρ: Spearman Correlation)

Model	ECFP	Fourier Feature	Cross attention (Compound)	Cross attention (Protein)	Results<br>Novel pair fold 0<br>Novel comp fold 0<br>Novel prot fold 0<br>(MSE/CI/PC/ρ)
Model 0		✓	✓	✓	0.914/0.450/-0.103/-0.104<br>1.174/0.612/0.233/ 0.244<br>0.349/0.749/0.494/0.426
Model 1	✓		✓	✓	0.938/0.413/-0.141/ -0.188<br>1.090/0.657/0.286/ 0.340<br>0.367/0.750/0.487/0.424
Model 2	✓	✓		✓	0.937/0.594/0.177/0.200<br>1.090/0.669/0.287/ 0.368<br>0.383/0.754/0.479/0.433
Model 3	✓	✓	✓		0.930/0.484/-0.028/-0.034<br>1.156/0.557/0.184/ 0.124<br>0.382/0.754/0.485/0.430
Model 4	✓	✓			0.998/0.444/-0.132/-0.118<br>1.115/0.596/0.198/0.210<br>0.435/0.725/0.479/0.383
Model 5 (where what exchange)	✓	✓	✓	✓	1.032/0.409/-0.229/-0.189<br>1.156/0.644/0.273/0.315<br>0.374/0.715/0.471/0.368
Model 6 (without 3D_comp)	✓		✓		0.979/0.472/-0.057/-0.080<br>1.176/0.560/0.131/0.206<br>0.374/0.715/0.471/0.368
Model 7 (without 3D_prot)	✓		✓		0.993/0.490/-0.014/-0.072<br>1.110/0.634/0.293/0.294<br>0.395/0.721/0.379/0.431
Model 8 (without 3D)	✓		✓		0.909/0.515/0.033/0.012<br>1.150/0.558/0.126/0.152<br>0.414/0.709/0.359/0.401
MulinforCPI	✓	✓	✓	✓	0.958/0.586/0.151/0.181<br>1.136/0.644/0.261/0.315<br>0.362/0.749/0.503/0.424

By integrating the Fourier feature, the model's learning capacity has been augmented, leading to a notable enhancement in performance. Consequently, the Pearson correlation metrics for all three configurations have demonstrated an upward trend, signifying the model's increased ability to generate predictions that closely align with the actual ground truth values.

In the absence of the cross-attention block of the Morgan fingerprint with PNA graph neural network, Model 0 exhibits a marginally superior performance compared to that of MulinforCPI. However, the performance in

the novel protein setting experiences a decline. On the other hand, when the cross-attention from the protein domain was omitted, the model exhibited poor performance on novel pair and novel compound tasks. Without two cross-attention blocks, the model's performance across all three tasks experienced a significant decline. When we swapped the positions of the *"where"* and *"what"* information at model 5, the model's performance noticeably deteriorated. This indicates that the atomic-level information is far more informative to the models than the global information.

To understand the importance of 3D (three-dimensional) information, we alternately removed the 3D information extractor from the compound's networks (Model 6) and the protein's network (Model 7), and from both of them (Model 8). To be more specific, we trained the PNA from scratch in Model 6, while only protein sequences were used to represent proteins. In two crucial tasks (Novel compound and Novel pair settings), all three models showed a significant decrease in performance across three metrics (CI/PC/ρ), indicating a notable decline in model generalization.

In conclusion, the collective integration of all components within MulinforCPI proves to be instrumental in achieving a robust performance in the final tasks.

2 Results form Metz and KIBA Dataset

Table 2: Result for novel hard pair in KIBA dataset.(MSE ↓ better, CI ↑ better, Spearman Correlation ↑ better, mean and standard deviation values were computed from five fold results' averages.)

Models	MSE	CI	Spearman correlation
DeepDTA	0.766(±0.077)	0.545(±0.023)	**0.126(±0.064)**
DeepConvDTI	0.752(±0.066)	**0.556(±0.033)**	0.115(±0.092)
TransformerCPI	1.024(±0.170)	0.514(±0.017)	0.039(±0.046)
GraphDTA (GINs)	2.428(±2.603)	0.542(±0.013)	0.118(±0.034)
HyperattentionDTI	0.963(±0.167)	0.537(±0.012)	0.103(±0.034)
PerceiverCPI	0.941(±0.153)	0.536(±0.038)	0.1(±0.106)
MulinforCPI (ours)	0.702(±0.096)	0.544(±0.026)	0.124(±0.072)
MulinforCPI (ours) Freeze 95%	**0.700(±0.081)**	0.542(±0.022)	0.118(±0.064)

Table 3: Restult for novel hard pair in Metz dataset.(MSE ↓ better, CI ↑ better, Spearman Correlation ↑ better, mean and standard deviation values were computed from five fold results' averages.)

Models	MSE	CI	Spearman correlation
DeepDTA	0.858(±0.073)	0.589(±0.034)	0.256(±0.099)
DeepConvDTI	0.872(±0.105)	**0.616(±0.035)**	**0.330(±0.099)**
TransformerCPI	1.17(±0.072)	0.532(±0.013)	0.093(±0.039)
GraphDTA (GINs)	2.374(±0.995)	0.591(±0.039)	0.258(±0.107)
HyperattentionDTI	1.043(±0.207)	0.612(±0.036)	0.321(±0.104)
PerceiverCPI	0.928(±0.079)	0.579(±0.042)	0.226(±0.118)
MulinforCPI (ours)	**0.751(±0.065)**	0.597(±0.027)	0.278(±0.079)
MulinforCPI (ours) Freeze 95%	0.783(±0.060)	0.577(±0.027)	0.223(±0.076)

The complexity of MulinforCPI necessitates a larger dataset for evaluating its performance in high-sparsity datasets compared to other competitors. Despite these challenges, the proposed MulinforCPI model exhibits promising outcomes, particularly in terms of the Mean Squared Error (MSE) metrics, where it outperforms other competing models. The model's ability to achieve superior results in this aspect reflects its efficacy in minimizing the discrepancies between the predicted and actual values, thus demonstrating its potential for accurate predictions. Furthermore, the MulinforCPI model showcases commendable performance in the context of Concordance Index (CI) and Spearman correlation metrics, positioning it competitively amongst its peers. While its performance may not be significantly superior to certain competing models, it remains a strong contender, indicating its capability to rank predictions.

3 Visualization for cross-domain experiment

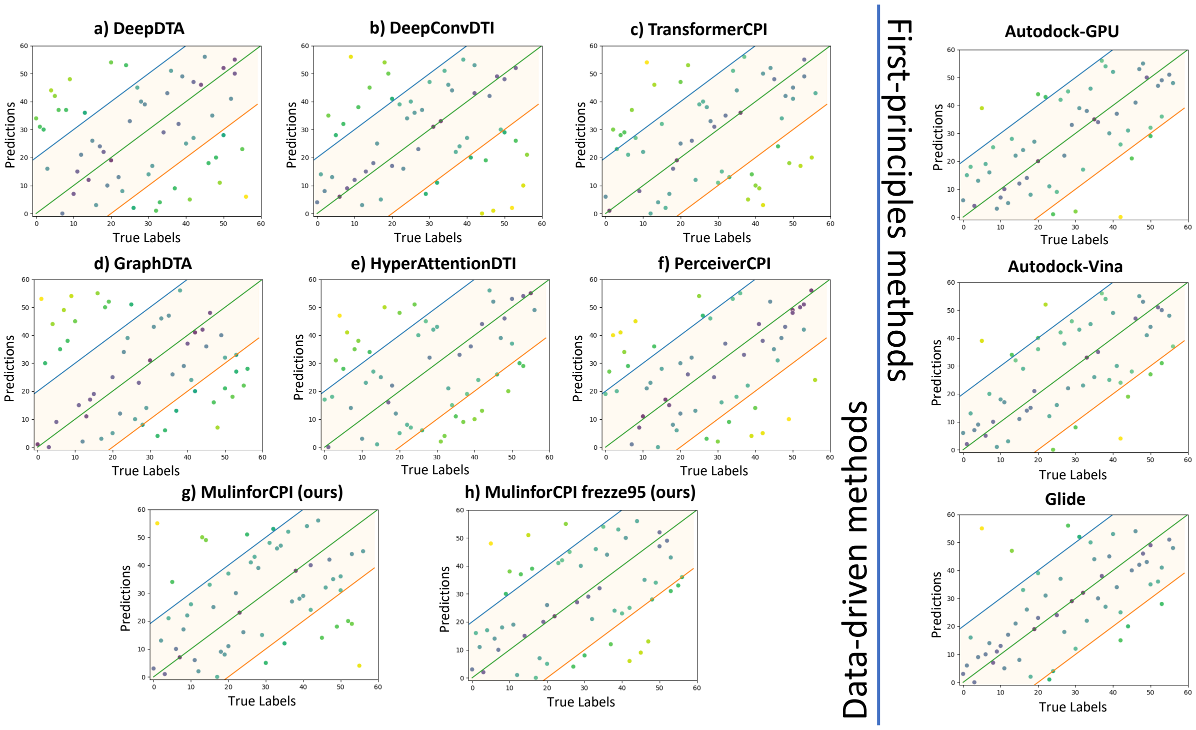

Figure 1: The cross-domain experiment evaluates the ranking predictions through a scatter plot visualization, comparing data-driven methods with docking-based methods. This analysis provides insights into the effectiveness of ranking prediction across different domains.

We visualized the predictions from all models, including the first-principles and data-driven methods, regarding the prediction rankings, as shown in Fig.1. This figure demonstrates that a significant portion of the ranking predictions generated by the various data-driven methods for the subset from CASF-2016 were arbitrary. Conversely, predictions derived from MulinforCPI and first-principles methods exhibit superior performance, exhibiting a pronounced linear relationship between the predicted and actual rankings. In three specific examples, MulinforCPI accurately predicted the ranks of the testing points. The intensity of the colors indicates the accuracy of the predictions, with lighter shades representing poorer predictions and darker shades indicating more accurate predictions.

4 Prediction visualization

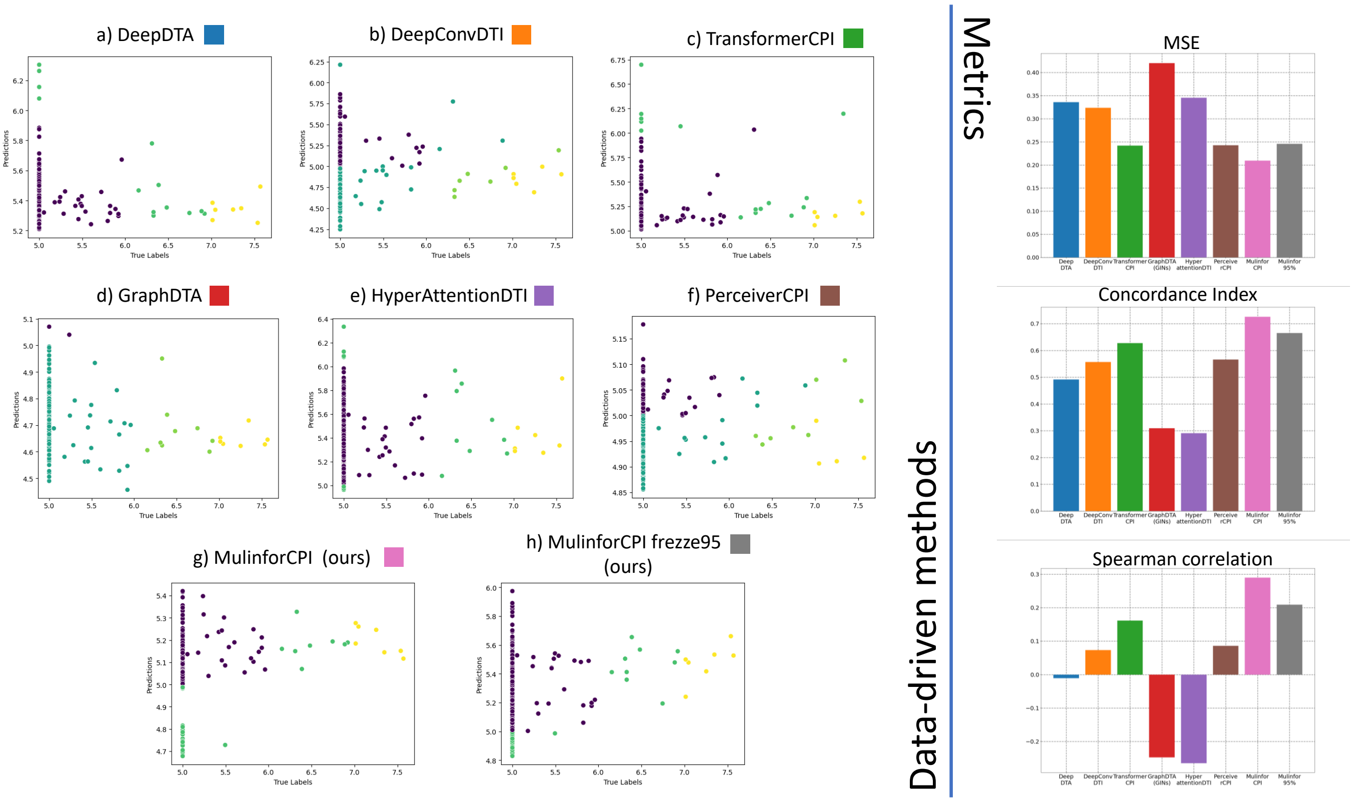

Figure 2: The scatter plot by predicted values for the third fold in novel pair setting.(MSE ↓ better, CI ↑ better, Spearman correllation ↑)

In this analysis, it becomes evident that the MulinforCPI model exhibits a discernible inclination towards aligning its predictions with the ascending values of the corresponding truth labels, as perceptible from the representation offered by the scatter plot 2. It appears that the current state-of-the-art (SOTA) models exhibit a tendency to generate predictions that show a high degree of randomness and deviate significantly from the corresponding labels. Furthermore, the MulinforCPI model demonstrates a remarkable superiority over state-of-the-art (SOTA) models across all three metrics, as clearly depicted in the accompanying Figure 2 in the metrics section. some baseline models exhibit negative Spearman Correlation values, which typically arise when the regression model is inadequately specified or when it is applied to data that violates the underlying assumptions of the model.

5 When reducing the level of test set difficulty

Table 4: The results cross-domain experiments when similarity threshold = 1 (MSE ↓ better, CI ↑ better, Spearman Correlation ↑ better).

Model	MSE	CI	Spearman correlation
DeepDTA	5.214	0.552	0.173
DeepConvDTI	4.946	0.586	0.277
TransformerCPI	4.725	0.617	0.349
GraphDTA (GINs)	6.275	0.541	0.113
HyperattentionDTI	5.168	0.569	0.189
PerceiverCPI	4.792	0.598	0.324
MulinforCPI (ours)	**3.929**	0.65	0.434
MulinforCPI (ours) Freeze 95%	4.277	0.643	0.426
Autodock-GPU	N/A	0.717	**0.620**
Autodock-Vina	N/A	0.711	0.608
Glide	N/A	**0.722**	0.614

In this experiment, we increased the threshold from 0.3 to 1, resulting in the removal of interactions that involved compounds or proteins appearing exactly in both the training and testing sets from the training set. By reducing the difficulty level of the testing set, a significant improvement in the performance of all deep learning models was observed. Specifically, Table 4 demonstrates that the performance of MulinforCPI exhibited notable enhancements. The mean squared error (MSE) decreased from 4.698 to 3.929, the confidence interval (CI) increased from 0.602 to 0.650, and the Spearman correlation improved from 0.297 to 0.434. These improvements demonstrate the positive impact of reducing the difficulty level of the testing set on the predictive capabilities of the deep learning models, particularly in the case of MulinforCPI.

6 Protein cut-off

Proteins are fundamental macromolecules that serve as crucial building blocks of life. They play a pivotal role in facilitating numerous biological functions owing to their highly specific molecular interactions. The role of a protein in any living organism is determined by the arrangement of its structural domains, and the size of a protein is a clear manifestation of this fact. The size of a protein in eukaryotes can range from just a few amino acids to many thousands. (Zhang, 2000)'s research findings indicate that the mean protein size in eukaryotic organisms is greater than that of bacterial and archaeal organisms, averaging approximately 400-500 amino acids with a corresponding molecular weight of roughly 45-55 kilodaltons. Besides, according to the length distribution of proteins across three primary datasets which is demonstrated in Fig 3, we have set a cut-off threshold of 500 amino acids. This length can effectively represent a substantial portion of most proteins, which can range in length from tens to thousands of amino acids. In certain instances, 500 amino acids could sufficiently cover the entire protein sequence. Additionally, cutting the protein sequence into smaller segments allows the computational algorithm to work more efficiently due to the computational insensitivity of both ESM-2 language model and Evoformer from Alphafold2 (Hie et al., 2022; Jumper et al., 2021). Furthermore, when dealing with a sizable dataset such as KIBA, which comprises approximately 80000 interactions, the dataset becomes exceedingly burdensome and unfeasible to load for proteins exceeding 500 amino acids in length.

Furthermore, we performed an experiment to investigate the model's capability in capturing meaningful information from proteins. In order to achieve this, we conducted an experiment on the Metz dataset using a novel hard pair setting. Specifically, we augmented the length of the training datasets. The results, presented in Table 5, demonstrate the efficacy of the MulinforCPI model in effectively extracting information from lengthy protein sequences, thereby enabling more accurate predictions for the Compound-Protein Interaction (CPI) task. Nevertheless, there exists a trade-off between performance and computational requirements. Although extending the length of training sequences enhances the model's learning capabilities, it also leads to a notable increase in storage requirements for storing the processed data, as indicated in Table 6. Additionally, the demand for memory significantly escalates in order to load the datasets, further highlighting the computational resources needed by the model.

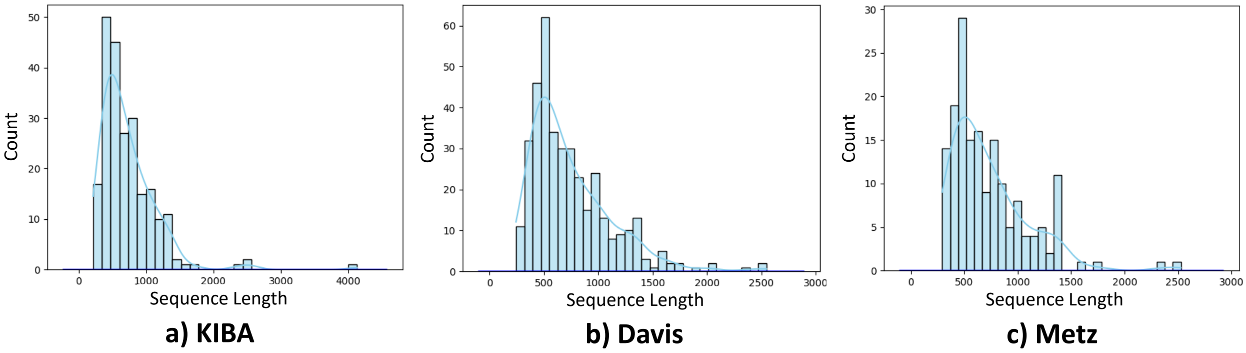

Figure 3: The protein length distribution of three training datasets. a) KIBA dataset, b) and c) Metz dataset.

Table 5: The comparison of MulinforCPI and for 500 amino acids and 750 amino acids cut off threshold for proteins. (MSE $\downarrow$ better, CI $\uparrow$ better, Spearman Correlation $\uparrow$ better)

Models	MSE	CI	Spearman Correlation
MulinforCPI (750aa)	**0.748(±0.057)**	**0.609(±0.041)**	**0.313(±0.103)**
MulinforCPI (500aa)	0.751(±0.064)	0.596(±0.027)	0.277(±0.078)

7 Model complexity

Utilizing a substantial number of parameters in a deep learning model presents several noteworthy advantages:

- Enhanced model capacity: More parameters enable the model to capture complex patterns and relationships in the data.
- Expressive power: Large parameter spaces provide the model with the flexibility to express a wide range of functions, enabling it to learn from diverse and intricate data patterns.
- Improved feature learning: Deep learning models can automatically learn hierarchical representations of the data through multiple layers.
- Enhanced generalization: large models can generalize well to unseen data, resulting in improved performance on validation or test sets.
- Transfer learning capabilities: By leveraging these pretrained models, practitioners can fine-tune specific layers or add additional layers to adapt the model to new tasks or datasets.

Nevertheless, it is crucial to acknowledge that large parameter counts entail computational and memory requirements. Training such models necessitates significant computational resources and prolonged training times. Additionally, large models may exhibit an increased susceptibility to overfitting, thereby demanding meticulous regularization and hyperparameter tuning.

In summary, the benefits of employing a substantial number of parameters in a deep learning model lie in its potential to augment model capacity, facilitate advanced feature learning, and foster superior generalization, thereby enabling the model to effectively address intricate tasks and attain heightened performance, subject to appropriate regularization and allocation of resources.

Table 6: The storage for Metz dataset.

Metz dataset	Storage (GB)
750aa	572
500aa	288

Table 7: The complexity of MulinforCPI and competitors.

Model	Complexity
DeepDTA (Öztürk, Özgür, & Ozkirimli, 2018)	1,981,185
DeepconvDTI (Lee, Keum, & Nam, 2019)	1,493,129
TransformerCPI(Chen et al., 2020)	416,730
GraphDTA (T. Nguyen et al., 2021)	1,297,505
HyperAttentionDTI(Zhao, Zhao, Zheng, & Wang, 2022)	2,308,561
PerceiverCPI (N.-Q. Nguyen, Jang, Kim, & Kang, 2023)	4,672,073
MulinforCPI (ours)	8,820,028

8 Model stability

In scientific research and experimentation, it is crucial to evaluate the stability of a deep learning model to ensure the reliability and reproducibility of the results obtained. One common approach to assessing stability is by conducting statistical analyses, such as calculating the standard deviation for several run times. Generally speaking, when the standard deviation is low, it indicates that the data points are close to the mean and there is less variability in the dataset. In the context of a model's stability, a low standard deviation suggests that the model's predictions or outputs are consistent and less likely to vary significantly.

Table 8: Results for 20 times run the MulinforCPI on first fold of Metz datasets.

Model	Runs	std (MSE)	std (CI)
MulinforCPI (ours)	20	0.028	0.015

9 Similarity check

To assess the similarity between two compounds, we employ the Tanimoto similarity metric from the rdkit library. For protein similarity calculation, we determine the ratio of aligned amino acids to the length of the protein sequence (in our case, we utilized a value of 500 for MulinforCPI) as shown in the following equation :

$$Similarityprot = \frac{\text{number of aligned amino acids}}{\text{total length of the sequence}} \quad (1)$$

Table 9: Similarity between training and test set of Davis dataset.

Davis dataset	Fold 0	Fold 1	Fold 2	Fold 3	Fold 4
Number of test protein	32	18	23	326	43
Number of training protein	410	424	419	116	399
Number of test compound	20	12	12	12	12
Number of training compound	48	56	56	56	56
Min similarity (Protein)	0.022	0.01	0.012	0.01	0.016
Max similarity (Protein)	0.522	0.484	0.522	0.7	0.7
Min similarity (Compound)	0.048	0.039	0.051	0.039	0.049
Max similarity (Compound)	0.483	0.483	0.444	0.429	0.416
Protein similarity (mean±std)	0.077±0.052	0.077±0.078	0.08±0.072	0.053±0.014	0.068±0.023
Compound similarity (mean±std)	0.138±0.056	0.132±0.05	0.133±0.055	0.135±0.05	0.137±0.049

Table 10: Similarity between training and test set of KIBA dataset.

KIBA dataset	Fold 0	Fold 1	Fold 2	Fold 3	Fold 4
Number of test protein	25	74	74	20	32
Number of training protein	176	152	154	205	192
Number of test compound	777	338	173	140	155
Number of training compound	337	543	1092	1181	1055
Min similarity (Protein)	0.022	0.014	0.016	0.02	0.014
Max similarity (Protein)	0.274	0.232	0.232	0.17	0.27
Min similarity (Compound)	0	0	0	0	0
Max similarity (Compound)	0.275	0.275	0.275	0.275	0.275
Protein similarity (mean±std)	0.063±0.03	0.057±0.013	0.057±0.014	0.069±0.024	0.065±0.05
Compound similarity (mean±std)	0.111±0.036	0.108±0.037	0.107±0.038	0.111±0.038	0.109±0.038

Table 11: Similarity between training and test set of Metz dataset.

Metz dataset	Fold 0	Fold 1	Fold 2	Fold 3	Fold 4
Number of test protein	17	77	22	19	34
Number of training protein	152	92	148	151	135
Number of test compound	562	208	102	108	113
Number of training compound	339	521	938	863	967
Min similarity (Protein)	0.022	0.016	0.026	0.016	0.022
Max similarity (Protein)	0.146	0.148	0.282	0.282	0.17
Min similarity (Compound)	0	0	0	0	0
Max similarity (Compound)	0.316	0.31	0.303	0.3	0.312
Protein similarity (mean±std)	0.06±0.013	0.057±0.013	0.075±0.051	0.07±0.056	0.062±0.016
Compound similarity (mean±std)	0.116±0.036	0.116±0.039	0.118±0.039	0.117±0.04	0.117±0.038

Table 12: Similarity between training and test set of cross-domain experiment

Cross-domain	Fold 0
Number of test protein	15
Number of training protein	170
Number of test compound	57
Number of training compound	1423
Min similarity (Protein)	0.008
Max similarity (Protein)	0.3
Min similarity (Compound)	0.012
Max similarity (Compound)	0.3
Protein similarity (mean±std)	0.072±0.068
Compound similarity (mean±std)	0.11±0.039

Table 13: Similarity between training and test set of Davis dataset with conventional 5-folds split technique.

Davis dataset	Fold 0	Fold 1	Fold 2	Fold 3	Fold 4
Number of test protein	82	78	80	103	99
Number of training protein	360	364	362	339	343
Number of test compound	14	14	14	14	12
Number of training compound	54	54	54	54	56
Min similarity (Protein)	0.01	0.016	0.01	0.016	0.012
Max similarity (Protein)	0.936	0.626	0.678	0.784	0.936
Min similarity (Compound)	0.039	0.052	0.051	0.039	0.048
Max similarity (Compound)	0.697	0.416	0.597	0.597	0.697
Protein similarity (mean±std)	0.067±0.041	0.067±0.041	0.066±0.041	0.064±0.029	0.068±0.042
Compound similarity (mean±std)	0.128±0.053	0.135±0.047	0.139±0.05	0.136±0.055	0.139±0.061

10 Result of MulinforCPI on decoy classification problem

Table 14: Statistic of Diverse Subset (7 Targets) from DUD-E database.

Targets (Diverse Subset)	Actives	Decoys
AKT1 (Serine/threonine-protein kinase AKT)	293	16,450
AMPC (Beta-lactamase)	48	2,850
CP3A4 (Cytochrome P450 3A4)	170	11,800
GCR (Glucocorticoid receptor)	258	15,000
HIVPR (Human immunodeficiency virus type 1 protease)	536	35,750
HIVRT (Human immunodeficiency virus type 1 reverse transcriptase)	338	18,891
KIF11 (Kinesin-like protein 1)	116	6,850

Table 15: Detailed enrichment factor analysis results for Diverse subsets from the DUD-E database.(EF1% / BEDROCα = 80.5).

Targets	DeepConvDTI	TransformerCPI	HyperattentionDTI	PerceiverCPI	MulinforCPI	Gold	Glide	Surflex	FlexX	Blaster
AKT1<br>(Serine/threonine-protein kinase AKT)	17.007/0.33	**37.415**/0.513	0.0/0.002	9.184	33.334/**0.533**	/0.42	-/0.24	-/0.05	-/0.11	29/-
AMPC<br>(Beta-lactamase)	0.0/0.003	0.0/0.0	0.0/0.003	0.0/0.0	0.0/0.005	-/0.04	-/**0.09**	-/0.00	-/0.04	**8**/-
CP3A4<br>(Cytochrome P450 3A4)	**14.67**/**0.232**	4.695/0.09	1.76/0.034	8.802/0.164	8.215/0.149	-/0.21	-/0.17	-/0.13	-/0.08	2/-
GCR<br>(Glucocorticoid receptor)	3.478/0.056	1.932/0.06	7.343/0.147	5.411/0.095	3.865/0.068	-/0.13	-/0.21	-/**0.30**	-/0.18	**9**/-
HIVPR<br>(HIV type 1 protease)	2.052/0.044	3.171/0.048	3.171/0.072	3.917/0.071	4.29/0.065	-/**0.30**	-/0.14	-/0.10	-/0.05	**5**/-
HIVRT<br>(HIV type 1 reverse transcriptase)	4.716/0.088	2.063/0.039	0.0/0.006	2.653/0.051	2.063/0.054	-/**0.42**	-/0.37	-/0.13	-/0.19	**7**/-
KIF11<br>(Kinesin-like protein 1)	2.574/0.072	0.0/0.002	0.0/0.002	2.574/0.064	3.432/0.088	-/0.55	-/**0.59**	-/0.12	-/0.08	**35**/-

11 Why CNN, not Transformer?

We empirically chose the 1D and 2D neural networks in the proposed network over the transformer architecture to extract information from proteins. It is important to note that the length of the atomic feature metric typically lies within the range of 4,000–5,000 elements. This range is often observed in scenarios involving approximately 500 amino acids and may increase further with longer protein sequences. Consequently, a significant allocation of computational resources is imperative to effectively handle such substantial lengths. This can be attributed to the parameter efficiency of CNNs, as they typically have fewer parameters than transformer models. CNNs are better at capturing spatial patterns and local dependencies, making them well-suited for tasks that involve matrices with fixed-length sequences. In contrast, transformers are particularly effective for handling sequential or temporal data. Moreover, transformers often have large parameter counts and require large datasets for effective training.

In this experiment, it is not feasible to directly apply the protein sequence at the atomic level to the transformer architecture due to its length, which ranges from 4000 to 5000 atoms. Therefore, we employed an additional 1DCNN to reduce the dimensionality before passing it to the transformer blocks. Furthermore, we substituted two CNN networks with transformer blocks. The performance of the transformer, as shown in Table 16 and 17, is competitive, albeit slightly lower than the performance achieved with CNN networks. Additionally, we observed that the training time is significantly longer when using the transformer architecture.

Table 16: The result for changing the CNN by transformer architecture for Metz dataset.(MSE $\downarrow$ better, CI $\uparrow$ better, Spearman Correlation $\uparrow$ better)

Models	MSE	CI	Spearman Correlation
MulinforCPI (Transformer)	0.803(±0.091)	**0.599(±0.036)**	**0.285(±0.103)**
MulinforCPI (ours)	**0.751(±0.064)**	0.596(±0.027)	0.277(±0.078)

Table 17: The result for changing the CNN by transformer architecture for Davis dataset. (MSE $\downarrow$ better, CI $\uparrow$ better, Spearman Correlation $\uparrow$ better)

Models	MSE	CI	Spearman Correlation
Novel pair settings			
MulinforCPI (Transformer)	0.549(±0.222)	0.546(±0.072)	0.093(±0.114)
MulinforCPI (ours)	**0.547(±0.256)**	**0.646(±0.05)**	**0.237(±0.061)**
Novel compound settings			
MulinforCPI (Transformer)	**0.678(±0.250)**	0.670(±0.031)	0.306(±0.053)
MulinforCPI (ours)	0.690(±0.275)	**0.679(±0.072)**	**0.317(±0.113)**
Novel Protein settings			
MulinforCPI (Transformer)	0.549(±0.222)	0.741(±0.019)	0.415(±0.014)
MulinforCPI (ours)	**0.488(±0.138)**	**0.756(±0.017)**	**0.439(±0.022)**

12 Implementation in detailed

12.1 Choosing K in K-means clustering

K-means clustering is known to perform better on low-dimensional data due to the challenges posed by high-dimensional spaces. In such spaces, the distance between points increases, and the concept of distance becomes less meaningful—an effect referred to as the "curse of dimensionality." To mitigate this, we initially applied PCA as a linear dimensionality reduction technique to reduce the dimension of the data points from 500 to 3. Subsequently, the K-means clustering technique was applied to cluster the data points based on this reduced dimensionality. Eventually, the data points were projected according to their cluster labels. We empirically chose K ∈ [5,10,15] and then selected the best Silhouette score as shown in Table 18. The Silhouette Coefficient is calculated using the mean intra-cluster distance and the mean nearest-cluster distance for each sample.

Table 18: Silhouette coefficient results in choosing the number of clusters.

Number of clusters	Silhouette score (protein) KIBA	Silhouette score (protein) Davis	Silhouette score (protein) Metz
**K=5**	**0.473**	**0.459**	**0.467**
K=10	0.371	0.380	0.367
K=15	0.409	0.364	0.331

12.2 Data processing

In terms of practical implementation, we have discovered the feasibility of generating predictions using Evolutionary Scale Modeling fold (ESMfold) during the training process. However, it is worth noting that the training speed of MulinforCPI is significantly slow due to not only the size of the ESMfold network but also the output which can be seen in Figure 4. Hence, we store all essential initial features from both compounds and proteins in tensors by the pickle (.pt) files prior to training the model. By employing this approach, we are able to minimize the time required for subsequent runs as the model is trained at a considerably accelerated pace.

In this process, we convert categorical features (3, 4, 10) into numerical vectors using a one-hot encoder, while the atom coordinates (7) are utilized to generate the distance map.

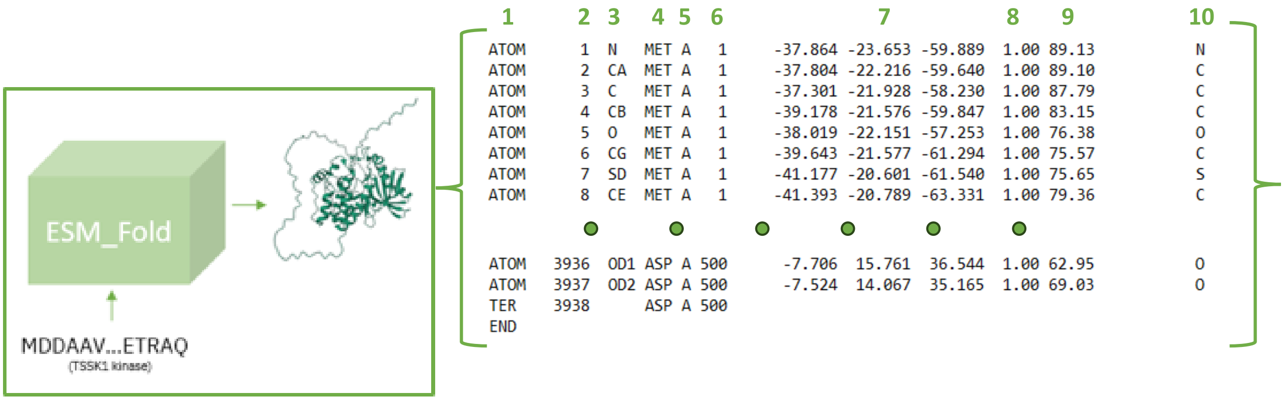

Figure 4: The output of ESM fold. The 3D fold contains 1) The Card, 2) Atom Number, 3) Atom Type, 4) Three-Letter Amino Acid Code, 5) Chain ID, 6) Residue Number, 7) Atom Coordinates, 8) Atom Occupancy, 9) Atomic Displacement Parameter, 10) Element.

12.3 Detailed Implementation MulinforCPI

Terms	Configuration
Optimizer	Adam
Learning rate	1.0e-4
Learning rate scheduler	WarmUpWrapper
Warmup steps	[150,50,30]
Wrapped scheduler	ReduceLROnPlateau
Factor	0.5
Patience	25
Min learning rate	1.0e-6

Block	Configuration
Compound network:	
Hidden dimension	200
Mid batch norm	True
Last batch norm	True
Bead_out batch_norm	True
Batch norm momentum	0.97
Readout hidden dim	200
Readout layers	2
Dropout	0.1
Propagation depth	7
Aggregators	[mean, max, min, std]
Scalers	[identity, amplification, attenuation]
Readout aggregators	[min, max, mean, sum]
Pretrans layers	2
Posttrans layers	1
Residual	True
Morgan embedding	nn.Embedding(2, 4)
Protein network:	
2DCNN network in	(inchannels=3, outchannels=4, kernelsize=5, stride=5, padding=2)
2DCNN network out	(inchannels=4, outchannels=1, kernelsize=5, stride=5, padding=2)
1DCNN network in	(inchannels=4000, outchannels=500, kernelsize=1)
1DCNN network out	(inchannels=65, outchannels=65*2, kernelsize=7, padding=7//2)
Cross-attention block:	
Compound cross attention block	MultiheadAttention(inputdim = 4, embeddim = 4,num_heads = 1)
Protein cross attention block	MultiheadAttention(inputdim = 20, embeddim = 20,num_heads=1)
Interaction:	
Compound out:	nn.Sequential(MLPs(400,128)
Protein out	nn.Sequential(MLPs(2048*4,128)
Interaction	nn.Sequential(MLPs(256, 1)

13 Data availability

The related links are as follows:
KIBA, Davis: https://github.com/kexinhuang12345/DeepPurpose
Metz: https://github.com/sirimullalab/KinasepKipred
BindingDB: https://github.com/IBM/InterpretableDTIP
DUD-E Diverse: http://dude.docking.org/subsets/diverse
QMugs: https://libdrive.ethz.ch/index.php/s/X5vOBNSITAG5vzM
CASF-2016: http://www.pdbbind.org.cn/casf.php

References

Chen, L., Tan, X., Wang, D., Zhong, F., Liu, X., Yang, T., ... Zheng, M. (2020). Transformercpi: improving compound–protein interaction prediction by sequence-based deep learning with self-attention mechanism and label reversal experiments. *Bioinformatics*, *36*(16), 4406–4414.

Hie, B., Candido, S., Lin, Z., Kabeli, O., Rao, R., Smetanin, N., ... Rives, A. (2022). A high-level programming language for generative protein design. *bioRxiv*, 2022–12.

Jumper, J., Evans, R., Pritzel, A., Green, T., Figurnov, M., Ronneberger, O., ... others (2021). Highly accurate protein structure prediction with alphafold. *Nature*, *596*(7873), 583–589.

Lee, I., Keum, J., & Nam, H. (2019). Deepconv-dti: Prediction of drug-target interactions via deep learning with convolution on protein sequences. *PLoS computational biology*, *15*(6), e1007129.

Nguyen, N.-Q., Jang, G., Kim, H., & Kang, J. (2023). Perceiver cpi: a nested cross-attention network for compound–protein interaction prediction. *Bioinformatics*, *39*(1), btac731.

Nguyen, T., Le, H., Quinn, T. P., Nguyen, T., Le, T. D., & Venkatesh, S. (2021). Graphdta: Predicting drug–target binding affinity with graph neural networks. *Bioinformatics*, *37*(8), 1140–1147.

Öztürk, H., Özgür, A., & Ozkirimli, E. (2018). Deepdta: deep drug–target binding affinity prediction. *Bioinformatics*, *34*(17), i821–i829.

Zhang, J. (2000). Protein-length distributions for the three domains of life. *Trends in Genetics*, *16*(3), 107–109.

Zhao, Q., Zhao, H., Zheng, K., & Wang, J. (2022). Hyperattentiondti: improving drug–protein interaction prediction by sequence-based deep learning with attention mechanism. *Bioinformatics*, *38*(3), 655–662.
